# Toward a serum-free, xeno-free culture system for optimal growth and expansion of hMSC suited to therapeutic applications

**DOI:** 10.1186/1753-6561-7-S6-P6

**Published:** 2013-12-04

**Authors:** Mira Genser-Nir, Sharon Daniliuc, Marina Tevrovsky, David Fiorentini

**Affiliations:** 1 Biological Industries, Kibbutz Beit Haemek, Israel

## Background

Human mesenchymal stem cells (hMSC) hold great promise as a tool in regenerative medicine and cell therapy. Application of hMSC in cell therapy requires the elaboration of an appropriate serum-free (SF), xeno-free (XF) culture system in order to minimize the health risk of using xenogenic compounds, and to limit the immunological reactions in-vivo. Besides the well-known disadvantages of serum, in comparison to a SF, XF culture system, serum also exhibits poor performance in the context of hMSC proliferation. In the present study, a novel SF, XF culture system for hMSC suitable for therapeutic applications was developed and evaluated. The SF, XF culture system includes specially developed solutions for attachment, dissociation, and freezing, as well as a culture medium, MSC NutriStem^® ^XF, that enables long-term growth of multipotent hMSC. Development of the SF, XF culture system was conducted on hMSC from a variety of sources: bone marrow (BM), adipose tissue (AT) and Wharton's jelly (WJ).

## Materials and methods

MSC NutriStem^® ^XF culture medium was examined in combination with MSC Attachment Solution (BI, 05-752-1) and either Recombinant Trypsin Solution (BI, 03-078-1) or MSC Dissociation Solution (BI, 03-075-1). The performance of MSC NutriStem^® ^XF was evaluated based on the following parameters: proliferation rate, viability, morphology, stemness (estimated from CFU-F), multilineage differentiation capability, and phenotypic surface marker profile [1].

### Cells

hMSC (passage 1-5) from a variety of sources: BM (Lonza, Promocell), AT (Promocell, ATCC), and WJ (ATCC, Prof. Mark Weiss - self isolation) were used in this study.

### Culture system

hMSC were cultured in a SF, XF expansion medium (MSC NutriStem^® ^XF, BI) on pre-coated dishes (MSC Attachment Solution, BI) or other media; commercial SF media (Invitrogen; SCT, Promocell), in-house serum-containing formulation (Prof. Mark Weiss). Cells were seeded at 5000-6000 viable cells/cm^2^, and harvested using either MSC Dissociation Solution (BI) or recombinant Trypsin Dissociation Solution (BI).

### Medium performance evaluation

Medium performance was evaluated by conducting a comparison of proliferation rate, cell morphology, multilinage differentiation potential into adipocytes, osteocytes, and chondrocytes, self-renewal potential and cell immunophenotype.

### Cell expansion

Cell proliferation was assessed by cell count using a trypan blue exclusion assay at each time point.

### Differentiation

hMSC expanded for 3-5 passages in MSC NutriStem^® ^XF were tested for maintenance of multilineage differentiation potential (into adipocytes, osteocytes, and chondrocytes) using in-house differentiation formulations. Undifferentiated control cells were cultured in MSC NutriStem^® ^XF. Cells were fixed and stained with Oil Red O, Alizarin Red/von Kossa, and Alcian blue/Masson's trichrome, respectively.

### CFU-F Assay

hMSC were seeded at low densities (10, 50, and 100 cells/cm^2^) in MSC NutriStem^® ^XF, cultured for 14 days, and stained with 0.5% crystal violet.

### Flow Cytometry

WJ-derived hMSC were cultured for five passages in MSC NutriStem^® ^XF, followed by immunophenotype evaluation by flow cytometry expression of CD73, CD90, CD105, HLA-ABC (positive), HLA-DR, and CD45 (negative).

## Results

**Figure 1:**
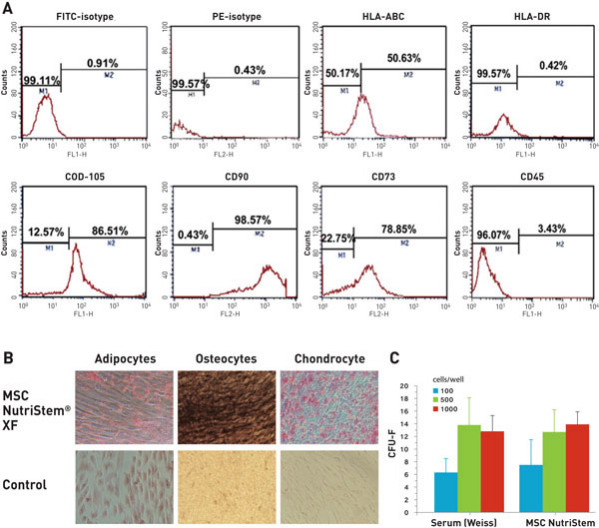
**hMSC Features after culturing in MSC NutriStem^® ^XF**. Characterization of hMSC-WJ expanded for 5 passages in MSC NutriStem^® ^XF and in-house serum-containing formulation. Immunophenotype using FACS analysis **(A)**, multilineage differentiation into adipocytes (Oil Red O), osteocytes (von Kossa), and chondrocytes (Masson's trichrome) **(B)**, CFU-F assay **(C)**. hMSC cultured in MSC NutriStem^® ^XF maintains the essential MSC characteristics; classical profile of MSC markers, multilineage differentiation, and self-renewal potential [1].

An optimized SF, XF culture system for hMSC was developed, composed of growth medium, MSC NutriStem^® ^XF, and all the required auxiliary solutions for the attachment, dissociation, and freezing of the cells. This SF, XF culture system for hMSC, supported optimal expansion of hMSC from a variety of sources, and exhibited superior proliferation compared with serum-containing media and commercially available SF media. hMSC expanded in the SF, XF culture system maintain their typical fibroblast-like cell morphology and phenotypic surface marker profile of CD73, CD90, CD105, HLA-ABC (all positive), or CD34, CD45, HLA-DR (all negative). hMSC differentiated efficiently after expansion in the developed SF, XF culture system into osteocytes, chondrocytes, and adipocytes. The self-renewal potential was maintained as well, demonstrated by a colony-forming unit fibroblast (CFU-F) assay (Figure 1).

## Conclusions

The use of serum is not an option from a regulatory point of view. A SF, XF culture system for hMSC was developed and enables long-term growth of multipotent hMSC suitable for therapeutic applications. The performance of MSC NutriStem^® ^XF medium was proved to be superior to serum-containing medium and commercially available SF media. MSC NutriStem^® ^XF medium supports long-term culture of hMSC from a variety of sources, while retaining the essential hMSC characteristics (fibroblast-like morphology, surface markers phenotype, multilineage differentiation, and self-renewal potential).The developed SF, XF culture system (MSC NutriStem^® ^XF medium, MSC Attachment Solution, either MSC Dissociation Solution or Recombinant Trypsin solution, and MSC Freezing Solution) supports the expansion of hMSC suitable for clinical applications.
